# Cleft Palate and Presurgical Orthopedics: A Systematic Review and Meta-Analysis of Intra-Arch Dimensions During the First Year of Life

**DOI:** 10.3390/jpm14121127

**Published:** 2024-11-29

**Authors:** Ana Rabal-Soláns, Carmen Mediero-Pérez, Rosa M. Yáñez-Vico

**Affiliations:** 1 School of Dentistry, Complutense University of Madrid, 28040 Madrid, Spain; 2 BIOCRAN, Craniofacial Biology and Orthodontics Research Group, School of Dentistry, Complutense University of Madrid, 28040 Madrid, Spain

**Keywords:** cleft palate, presurgical orthopedics, intra-arch dimensions, craniofacial anomalies

## Abstract

**Background**: This systematic review and meta-analysis aimed to investigate the effects of presurgical orthopedics (PSO) on maxillary arch dimensions in infants with cleft lip and palate during the first year of life. **Methods**: The review was conducted following PRISMA guidelines. A comprehensive electronic search was performed in MEDLINE, Embase, Cochrane, Scopus, and Google Scholar databases, supplemented by manual searching. Two reviewers independently conducted study selection, data extraction, quality assessment, and risk of bias evaluation. **Results**: Five studies were included in the meta-analysis. Quantitative analysis was performed based on the primary outcomes. The estimate was calculated using a random-effects model and z distribution (95% confidence interval (CI)). The results showed similar alveolar cleft widths (mean difference, −3.06; 95% CI, −8.03 to 2.70, *p* = 0.30, I2 = 99%) with clinical differences in favor of PSO, and comparable posterior cleft widths (mean difference, −0.88; 95% CI, −2.06 to 0.30, *p* = 0.14, I2 = 89%) with and without PSO in CLP babies. **Conclusions**: This meta-analysis found no statistically significant effects of presurgical orthopedic treatment on maxillary arch dimensions in infants with cleft lip and palate during the first year of life. Further high-quality randomized controlled trials are needed to definitively establish the efficacy of PSO.

## 1. Introduction

Cleft lip and palate (CLP) are a significant congenital orofacial anomaly that can lead to various secondary complications, which may result in negative outcomes that significantly affect their overall health and well-being [1]. This condition typically requires surgical intervention within the first year of life to address both functional and aesthetic concerns [2]. The primary goal of treatment is to achieve anatomical rehabilitation, including the division of the maxilla, to promote balanced maxillary growth and prevent dental arch deformities [3,4].

Several studies have outlined various early treatment protocols, many of which employ presurgical orthopedics (PSO) as the primary course of action [5,6]. PSO involves the use of various intraoral devices aimed at repositioning displaced tissues secondary to the cleft deformity prior to lip and nose repair [7]. Despite its widespread implementation in approximately half of CLP treatment protocols, recent studies have yielded inconsistent results, leading to ongoing debate regarding its efficacy [5,7,8]. The effects of PSO have been evaluated from multiple perspectives, including speech development [9], facial aesthetics [10,11], nasal deformity [12,13], facial growth [14], caregiver satisfaction [15], feeding outcomes [16], dental arch width [17], and short- and long-term effects [4,8]. Similarly, various protocols implemented across different hospitals have been analyzed and compared [18], and treatment outcomes related to alveolar morphology and maxillary growth and development have been studied in children who underwent various surgical protocols [19]. Some authors argue that its main advantage lies in establishing an appropriate transverse relationship of the maxilla and potentially reducing the need for future surgeries [20,21]. However, other authors contend that PSO may interfere with transverse maxillary growth, which is considered a significant disadvantage [22]. Therefore, the present systematic review and meta-analysis aimed to critically evaluate the efficacy of presurgical orthopedic protocols in patients with cleft lip and palate. Specifically, we seek to quantify the impact of PSO on maxillary arch shape and dimensions in infants with CLP compared to those who did not receive PSO treatment.

## 2. Materials and Methods

This systematic review was registered with PROSPERO (International Prospective Register of Systematic Reviews; www.crd.york.ac.uk/prospero) with registration number CRD4202230341. The present systematic review was conducted in accordance with the guidelines of the Cochrane Handbook for Systematic Reviews of Interventions [23] and was reported according to the criteria established in the protocol of the PRISMA declaration (Preferred Reporting Items for Systematic Review and Meta-analysis) [24].

### 2.1. Eligibility Criteria

The PICOS question for this systematic review was as follows: “Did presurgical orthopedics in patients with CLP affect intra-arch dimensions before surgery?” The population included patients aged 0–12 months with CLP, unilateral or bilateral, complete or incomplete, without associated syndrome. The intervention involved studies with a total sample size of *n* ≥ 30 in which PSO was performed with fixed or removable appliances. The comparison was made with patients who had CLP, unilateral or bilateral, complete or incomplete, without presurgical orthopedics. The outcome measures included variables that determine inter-arch width and approximation of the segments. The study design included randomized clinical trials (RCT), non-randomized clinical trials (NRCT), quasi-randomized trials (QRCT), and controlled before-and-after (CBA) studies. Excluded were animal studies, non-comparative studies (case reports, case series, opinion pieces, letters to the editor), systematic reviews and meta-analyses, studies based on incomplete text or results, studies in which comparisons were not made, studies in which the effects of PSO were compared with different surgical techniques for primary lip closure, and studies in which patients presented some syndromes associated with CLP in which only the nasal stent and/or facial aesthetics and/or speech and/or feeding and its consequences were evaluated, not the PSO treatment itself; studies in which only facial growth was evaluated as a consequence of PSO treatment and those in which data were obtained through surveys of parents of children with CLP were also excluded.

### 2.2. Search Strategy

**Figure 1:**
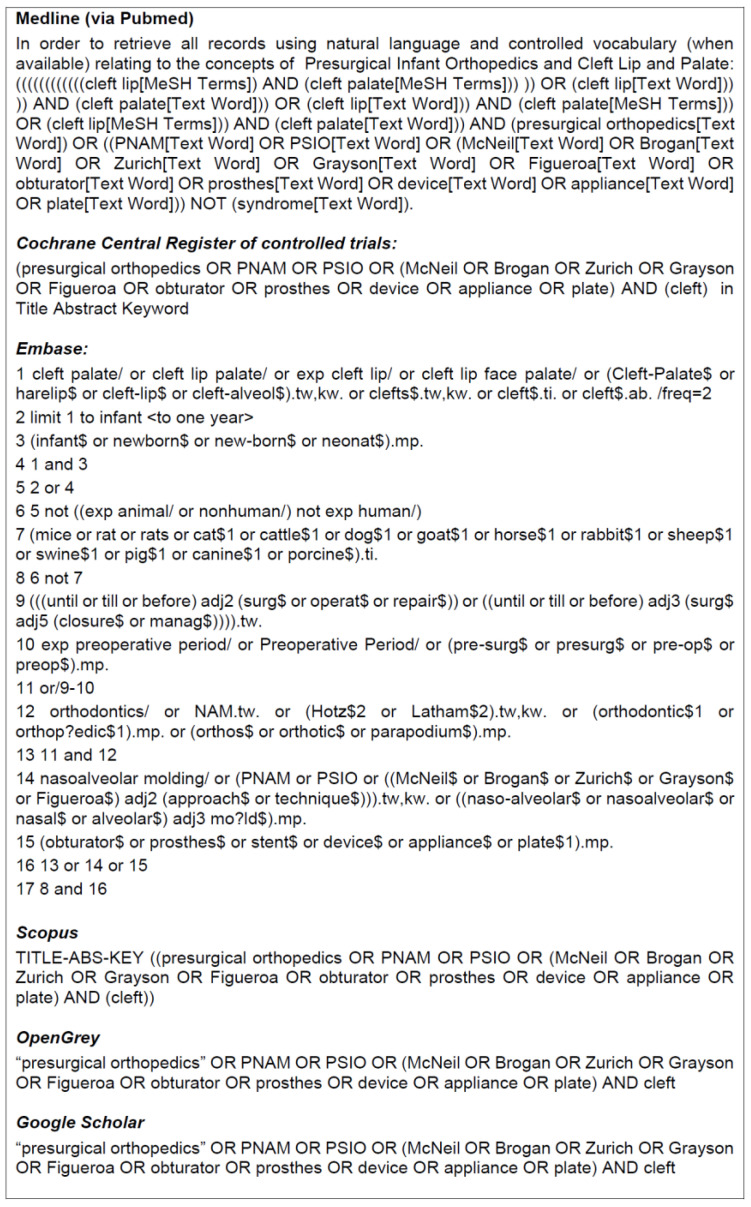
Searches strategies.

A comprehensive search strategy was developed and implemented across four electronic databases: MEDLINE (accessed via PubMed), Cochrane, Embase, and Scopus. The search terms included variations of “presurgical orthopedics” and “cleft,” combined using Boolean operators (AND, OR) (Figure 1). To identify additional relevant studies, the authors searched grey literature sources (Google Scholar and OpenGrey) and conducted manual searches of articles related to, and cited by, the included studies, to identify relevant studies. Studies published in any language since 1990 were considered eligible if they provided data on inter-arch dimensions before and after surgery comparing PSO and non-PSO groups. All studies published up until November 2023 were included in the analysis.

Two of the researchers (R.-S.A., M.C.) independently conducted the study selection process without blinding to study affiliations or authorships. Disagreements were resolved through discussion, with a third researcher (Y.-V.R.) serving as the final arbiter when necessary. The studies were selected based on their title and abstract. In the second stage, the full text of the potentially eligible studies was analyzed. Data extraction was performed using a standardized, pre-piloted form. The following information was extracted from each included study: (a) citation details, (b) publication date, (c) study design, (d) sample size, (e) inter-arch dimensions (anterior cleft and alveolus width variables and posterior cleft and alveolus width variables), (f) methodology used and age of patients, and (g) principal results.

### 2.3. Risk of Bias Assessment

The quality assessment of included studies was conducted in accordance with the Cochrane Handbook for Systematic Reviews of Interventions [23]. Two authors (R.-S.A., M.C.) independently evaluated the risk of bias across the following domains: random sequence generation (selection bias), allocation sequence concealment (selection bias), blinding of participants and personnel (performance bias), blinding of outcome assessment (detection bias), incomplete outcome data (attrition bias), and selective reporting (reporting bias). Disagreements were resolved through discussion or consultation with a third author (Y.-V.R.). The inter-rater agreement between the reviewers was assessed using κ statistics, which ranged from 0.86 to 0.92. Scoring criteria were established a priori through consensus among all reviewers.

### 2.4. Statistical Analysis

A quantitative analysis was performed for the primary outcomes using a random-effects model. Effect estimates and 95% confidence intervals (CIs) were calculated using the z distribution. Results are presented in forest plots, including global effect measures and CIs. The relative weight of each study was estimated using standard meta-analytic techniques. The percentage of variability of the estimated effect, which can be attributed to the heterogeneity of the true effects (I2), and the chi-square test were performed.

## 3. Results

### 3.1. Literature Search

**Figure 2:**
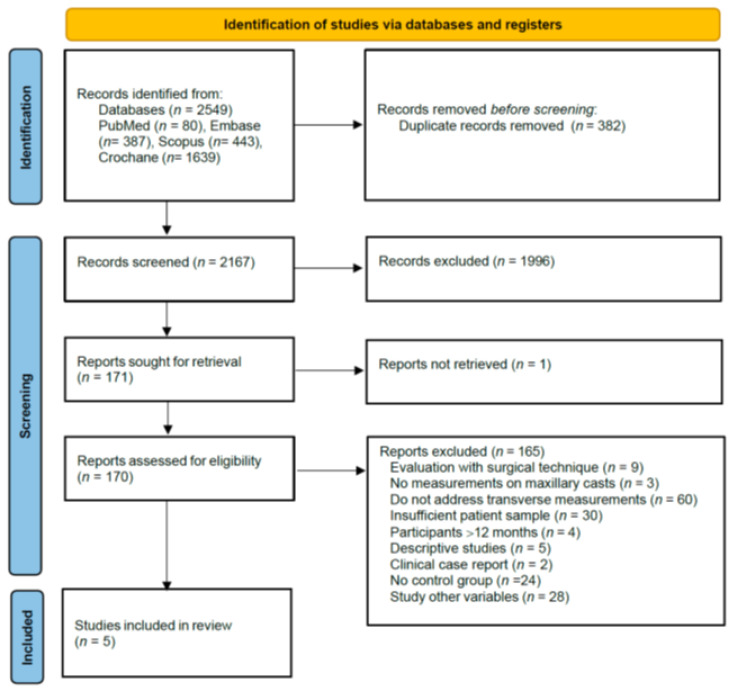
PRISMA flow diagram of study selection process. Adapted from PRISMA 2020.

A comprehensive literature search identified 2549 potentially relevant articles across multiple databases (PubMed *n* = 80; Embase (*n* = 387); Scopus (*n* = 443); Cochrane (*n* = 1639), and one additional article was identified through manual searching. After title and abstract screening, 170 studies underwent full-text review. Of these, 165 were excluded based on predefined criteria. The detailed study selection process is illustrated in the PRISMA flow diagram (Figure 2). The remaining five articles [5,25,26,27,28] that fulfilled the eligibility criteria were included in this review.

### 3.2. Study Characteristics

The five included studies comprised a total sample of 365 neonates, of whom 145 had cleft lip and/or palate and received presurgical orthopedics (PSO) before surgery in their first year of life. All studies focused on unilateral complete cleft lip and palate (UCCLP). No studies on bilateral cleft lip and palate met the inclusion criteria. The studies were distributed across three publication timelines: one in the first decade (1990–2000) [25], one in the second decade (2001–2010) [26], and three in the third decade (2011–2020) [5,27,28]. Although most studies measured the maxillary casts of patients [5,25,26,28], one study used 3D digitized models [27]. The outcomes variables analyzed included anterior cleft width [5,27,28], intercanine distance [5,27,28], intertuberosity distance [5,25,26,27,28], posterior cleft width [5,26,27], and transverse maxillary measurements, which were all obtained through anthropometric measurements.

**Figure 3:**
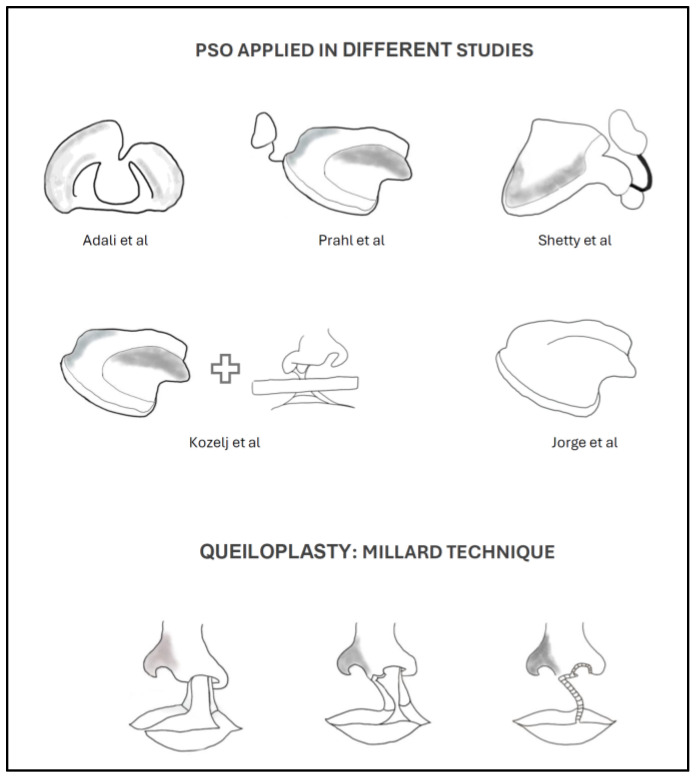
Different protocols from presurgical orthopedics (PSO) and lip closure among the analyzed studies.

The design and protocol for presurgical orthopedics (PSO) varied among the analyzed studies (Figure 3). Adali et al. (2012) [5] used active PSO plates from birth, consisting of two overlapping acrylic components controlled by a U-shaped spring, and at three months of age, surgical lip repair was performed using a modified Millard technique with a single-layer vomer flap. In another study [25], presurgical treatment comprised a thin, passive acrylic plate and slim adhesive tape fixed to the lip segments to bring them slightly together. The plate was inserted on the 20th day after birth and used until lip surgery, which occurred between the fifth and seventh months.

Similarly, PSO was performed using passive plates fabricated on a plaster cast and consisted of compound soft and hard acrylic, starting within two weeks after birth, and lip surgery was performed according to the Millard technique [26]. However, a different protocol was followed [28], where patients wore PNAM, and insertion varied (before the first month of age, 1 to 6 months, and 6 to 12 months), followed by a modified rotation advancement cheiloplasty at the completion of PNAM. Finally, in one study [27], a treatment protocol different from the others was employed, where patients wore a Hotz plate from birth, and surgical lip repair was performed using a modified Millard, Veau-Grob, or Celesnik technique. A summary of the main features of included studies is provided in Table 1.

### 3.3. Risks of Bias

**Figure 4:**
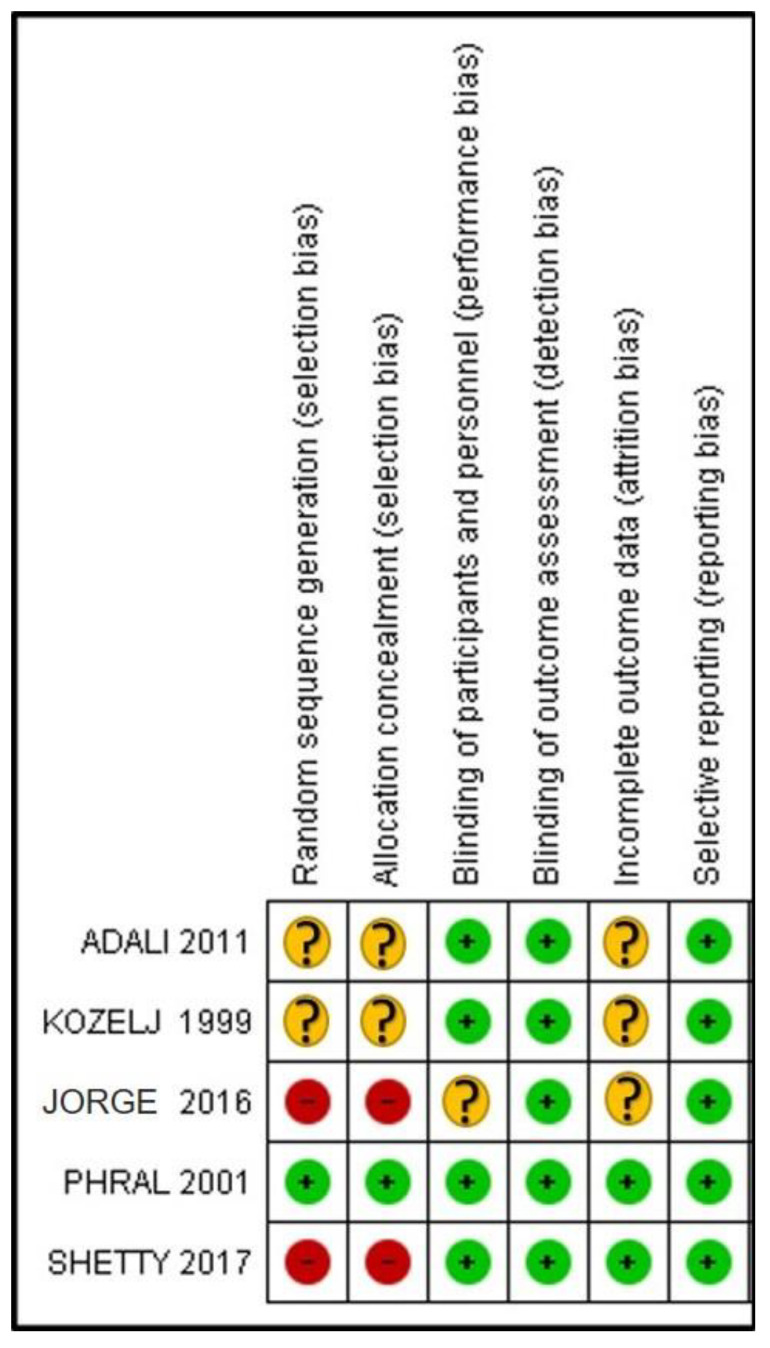
Results of the risk of bias assessment [5,25,26,27,28]. 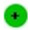 low risk of bias 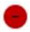 high risk of bias 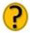 unclear risk of bias.

The risk of bias assessment, conducted according to the Cochrane Handbook for Systematic Reviews of Interventions, is summarized in Figure 4. The estimated potential risk of bias was low for one study [26], moderate for two [5,25], and high for two studies [27,28]. The main sources of bias were related to random sequence generation and allocation concealment (selection bias).

### 3.4. Quantitative Synthesis

**Figure 5:**
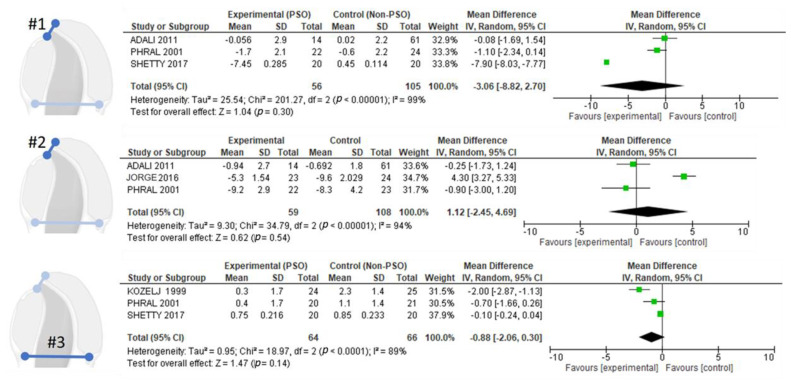
Forest plot of the changes produced in; #1 (alveolar cleft width) between T1-T2 (approximately birth vs. 3 months dimensions); #2 (alveolar cleft width) between T2-T3 (approximately 3–12 months dimensions); #3 (posterior cleft width) [5,25,26,27,28].

After a comprehensive analysis of the variables used across studies, four were selected for meta-analysis: to analyze the changes produced in #1 (alveolar cleft width) between T1–T2 (approximately birth vs. 3 months dimensions) [5,26,28] and #2 (alveolar cleft width) between T2–T3 (approximately 3–12 months dimensions) [5,26,27] and #3 (posterior cleft width) [25,26,28]. Due to high heterogeneity among studies, a random-effects model was applied for meta-analysis. The results showed no statistically significant differences between presurgical orthopedics (PSO) and control groups for any of the analyzed outcomes (Figure 5).

Regarding the changes in alveolar cleft width between T1–T2 (approximately birth vs. 3 months dimensions (#1), three studies [5,26,28] showed an effect in favoring presurgical orthopedics (PSO), although only one study [28] showed a clear effect favoring the experimental group with respect to the control group (−7.90, 95% CI: −8.03, −7.77). However, the overall effect was not statistically significant (*p* = 0.30). For the changes produced in alveolar cleft width between T2–T3 (approximately 3–12 months dimensions) (#2), one study [27] showed a clear effect favoring the control group (95% CI: 3.27–5.33). The changes in posterior cleft width (#3) found three studies showed effects favoring PSO: Kozelj et al. (1999) (95% CI: −2.87 to −1.13) [25], Phral et al. (2001) (95% CI: −1.66, 0.26) [26] and Shetty et al. (2017) (−0.10, 95% CI: −0.24, 0.04) [28]. The global contrast test results were not statistically significant (*p* = 0.14).

## 4. Discussion

The earliest intervention for patients with CLP usually begins within the first weeks of life, with the goal of improving both the skeletal and soft tissue anatomy, as well as enhancing the alignment of the alveolar segment prior to lip repair. Additionally, it aims to improve nutrition and aesthetics, increase columellar length, and achieve nostril symmetry [29].

According to our knowledge, no previous meta-analysis has explored the short-term effects of PSO treatment in newborns with CLP. Therefore, the purpose of this study was to analyze the data from studies published to date that assessed changes in the maxillary arch of CLP patients during the first year of life after PSO treatment, compared to CLP patients who did not receive PSO treatment.

PSO treatment is widely used in CLP referral centers. As indicated by a recent study [30], 67.8% of centers incorporate PSO treatment in their protocol, a value that has increased from the 57% reported in a previous study [31]. Nasoalveolar molding (NAM) is the most commonly used technique, with values increasing from 60.8–83.3%, while the Latham technique accounts for 24.3% [30,31].

The primary goal of this systematic review and meta-analysis was to assess whether the implementation of PSO prior to surgery had an impact on the inter-arch dimensions during the first year of a patient’s life. The examination of the selected studies did not uncover any clear positive, negative, or neutral patterns in the transverse dimensions of the cleft or alveolar arch, as the results did not indicate a statistically significant difference. Despite the lack of statistical differences identified through the meta-analytic study, a thorough examination of the results revealed clinically significant differences in favor of the use of PSO. These findings could be attributed to the varying clinical approaches to treating CLP patients, particularly during their first year of life.

In Adali’s study [5], researchers used a PSO consisting of two acrylic plates connected by a U-shaped metal spring. This device allowed the execution of all types of movements, and external support was not required to improve its retention. Other investigations [25,26] used a passive acrylic plate covering the alveolus and hard palate and mimicking the typical alveoli in the cleft area. Furthermore, a small nasal extension may be used. Additionally, an active acrylic mold that was modified sequentially with the intention of relocating the oronasal structures to their normal position was also employed [28]. The NAM has been compared with newer PSO devices, such as the DynaCleft, with findings indicating that both systems reduce the width of the cleft in patients with UCLP and improve nasal symmetry [32]. Differing from the aforementioned authors, Jorge et al. [27] utilized a Hotz plate as a PSO treatment. Despite being a widely used technique, NAM therapy achieved better results in reducing the width of the anterior segment, as well as a lesser increase in the depth of the cleft. Although there is a long history of treating patients with CLP, there is still no consensus on treatment protocols [31]. Another issue is the significant variability among treatments, which can lead to confounding factors when obtaining results. Recent studies have explored whether improvements in treatments have resulted from the standardization of protocols.

The treatment approaches employed for CLP vary significantly from hospital to hospital, as demonstrated by Weinfeld et al. (2005) [31], who illustrated a wide range of protocols. Notably, 33.3% of the centers performed definitive lip surgery in patients with UCLP before the age of 3 months. Most of the centers studied (65.9%) performed lip surgery between 3–6 months of life to establish lip competence by unifying the orbicularis muscle, which is critical for normal feeding and speech functions. Only 0.7% of the centers performed lip surgery after 6 months of age. Shetty et al. (2017) [28] was the only study that subdivided the patient groups into two subgroups that underwent lip surgery between 6–15 months of age.

Regarding the surgical technique used, there is greater consensus among centers. 82.4% of the centers used the Millard rotation-advancement technique or its modifications, in agreement with the selected studies, which also utilized this surgical approach [5,25,26,28]. Only one study [27] also used the Veau-Grob, Celesnik, and Spina techniques.

The main issue that arises in the surgical aspect is the frequent limitation in interpreting the results, which leads to an important bias. This is because patients were operated on by different surgeons who used various surgical techniques simultaneously [10,33]. To eliminate this bias, the design of this review aimed to eliminate it in the studies. Although many authors consider the use of different surgeons and lip and palate closure techniques within the same team to be a positive aspect, it is essential to homogenize the sample for the interpretation of results. To avoid this bias in the meta-analysis data, the authors decided to exclude all studies that evaluated the results of PSO together with the surgical technique used.

The most significant limitation of the literature review in relation to PSO is the lack of studies with large patient samples due to the low prevalence of CLP. Despite this low prevalence, advancements in pediatric healthcare have led to a reduction in mortality rates associated with congenital abnormalities. Nevertheless, birth defects remain a significant and escalating concern that requires a multidisciplinary approach [34].

Despite the aforementioned limitations, this systematic review and meta-analysis have considerable strengths due to the strict protocol that was followed, which was registered in PROSPERO and followed the guidelines of evidence-based medicine with limited inclusion criteria regarding the type of sample and follow-up period. Additionally, all steps were performed in duplicate to minimize individual bias. To improve future studies, it is suggested that more homogeneity in measurement times and uniformity in measurements be achieved to obtain more inter-arch information that can be compared.

## 5. Conclusions

The findings of this study provide valuable insights into the use of PSO in the management of cleft lip and palate in infants. The study was limited to the available literature and further research is needed to confirm the findings and validate currently used treatment protocols. The results of the study suggest that, within the limitations of the included studies, the use of PSO did not produce statistically significant changes in the dental arches during the first year of life in patients with CLP. However, clinically significant changes were observed in alveolar cleft width in patients who underwent PSO before surgical lip repair. Future studies should also evaluate the standardization of child treatment protocols.

## Figures and Tables

**Table 1:** Characteristics of the included studies.

Authors	Sample (n)	Variables	Treatment Protocol	Results
Kozelj, V. 1999 [17]	PSO n = 24 No PSO n = 25 Non Cleft n = 25	Tr-TI: Point at the bottom of the palatine foalveola below the tuberosityon the right and left sides.	Average at insertion: 8.9 d. Average at the conclusion: 186 d. Average age first cast (T1): PSO 9.8 d. No PSO at birth. Average age second cast pre-lip repair ( T2): PSO 193 d. No PSO 6 m.	After PSO, the upper oral cavity was remodeled and slightly enlarged; there was a lesser difference from the noncleft at 6 months than at birth. The cleft in the alveolus reduced significantly, and the position of the incisive point improved. The No PSO group had no remodeling, and the growth dynamics were similar to the non-clef, the dimensional differences from the normal reamined the same as birth.
Prahl, C. 2001 [18]	PSO n = 24 No PSO n = 25	P’L’ Alveolar cleft width: distance between point P and L. C- C’ intercanine point distance, distance between point C and C’; T-T’: intertuberosity point distance, distance between point T and T’. t-t’: margins of the posterior cleft at the tuberosity points level	Maxillary impresión PSO- No PSO ( T1): 2 wk of age. Maxillary impresión PSO- No PSO ( T2): 15 wk of age. Maxillary impresión PSO- No PSO ( T4): 48 wk of age.	Before lip closure, alveolar, midpalatal and posterior cleft width reduced significantly more in PSO than No PSO. After lip closure, the alveolar cleft width reduced significantly more in No PSO. PSO only has a temporary effect on maxillary arch dimensions that does not last beyond surgical soft palate closure.
Nazan Adali, 2012 [3]	PSO n = 14 No PSO n = 61	A-A1: Alveolar cleft width C-C1: Anterior arch witdh G-G1: Posterior cleft witdh E-E1: Posterior arch witdh	Mean age at birth impression (T1): PSO 6.2 d. No PSO 8.5 d. Mean age at pre-lip repair impression (T2): PSO 3 m 22 d. No PSO 3 m 16 d. Mean age at pre-palate repair impression (T3): PSO 7 m 18 d. No PSO 6 m 14 d	Presurgical orthopedics produced no statistically significant mean change in any archform variable when compared with the No PSO group. The difference in the mean reduction in the alveolar cleft width between the groups was 0.69 mm (95% IC, -0.89 to 2.28 mm, p = 0.52). Lip repair produced greater change in archform tahn did PSO, reducing the mean alveolar cleft width by 4.45 mm ( 95% IC, 3.53 to 5.37 mm; p = 0.001)
Jorge PK. 2016 [19]	PSO n = 23 No PSO n = 24	P-P’: anterior cleft width: Distance between right and left anterior cleft edges. C-C’: intercanine distance: Distance between the right and left lateral sulci of the alveolar ridge crest. T-T’: intertuberosity distance:	Maxillary digital cast PSO- No PSO (T1): Before surgical lip repair. Maxillary digital cast PSO_ No PSO ( T2): 1 year of age.	The intercanine distance decreased in No PSO group, indicating that the maxillary segments became repositioned at the anterior portion of dental arch after lip repair. At T2, the anterior cleft width, the intercanine distance and the anteroposterior cleft distance were all smaller in No PSO group than in Group I. The increased narrowing of the anterior and posterior cleft widths in No PSO group compared with with PSO group indicates that the use of PSO prevented excessive approximation of maxillary segments after lip repair.
Shetty, V. 2017 [20]	PSO n = 60 No PSO n = 60	ISD: Intersegment distance: Measurements between the tangents to the most medial curvature at the center of the ridges. ICW: Intercanine width : Distance between the canine grooves or lateral sulcus points (the point at which the lateral sulcus crosses the crest of the alveolar ridge) PAW: Posterior arch width: Distance between the retromolar points (posterior limit of tuberosity)	PSO started in group I at 1 month until 6 months of age. PSO started in group II between 1–6 months and last for a minimum of 3 months. Maxillary impresión PSO (T1): At the time of initiation. Maxillary impresión PSO (T2): On completion of NAM. Maxillary impresion No PSO (T1): First visit. Maxillary impression No PSO (T2): Before cheiloplasty.	ISD reduced significantly in PSO group but increased in control group. ICW did not show significant changes between two groups. The ISD reduced following PNAM improves arch symmetry and stability, and thus may prevent arch collapse in the long term.

## Data Availability

Not applicable.
